# Domestic and sexual violence against patients with severe mental illness

**DOI:** 10.1017/S0033291714001962

**Published:** 2014-09-04

**Authors:** H. Khalifeh, P. Moran, R. Borschmann, K. Dean, C. Hart, J. Hogg, D. Osborn, S. Johnson, L. M. Howard

**Affiliations:** 1 Division of Psychiatry, Faculty of Brain Sciences, UCL (University College London), UK; 2 Health Service and Population Research Department, Institute of Psychiatry, King's College London, UK; 3 School of Psychiatry, UNSW and Justice Health and Forensic Mental Health Network, NSW, Australia

**Keywords:** Mental illness, psychosis, physical violence, sexual violence, victim

## Abstract

**Background:**

Domestic and sexual violence are significant public health problems but little is known about the extent to which men and women with severe mental illness (SMI) are at risk compared with the general population. We aimed to compare the prevalence and impact of violence against SMI patients and the general population.

**Method:**

Three hundred and three randomly recruited psychiatric patients, in contact with community services for ⩾1 year, were interviewed using the British Crime Survey domestic/sexual violence questionnaire. Prevalence and correlates of violence in this sample were compared with those from 22 606 general population controls participating in the contemporaneous 2011/12 national crime survey.

**Results:**

Past-year domestic violence was reported by 27% *v.* 9% of SMI and control women, respectively [odds ratio (OR) adjusted for socio-demographics, aOR 2.7, 95% confidence interval (CI) 1.7–4.0], and by 13% *v.* 5% of SMI and control men, respectively (aOR 1.6, 95% CI 1.0–2.8). Past-year sexual violence was reported by 10% *v.* 2.0% of SMI and control women respectively (aOR 2.9, 95% CI 1.4–5.8). Family (non-partner) violence comprised a greater proportion of overall domestic violence among SMI than control victims (63% *v.* 35%, *p* < 0.01). Adulthood serious sexual assault led to attempted suicide more often among SMI than control female victims (53% *v.* 3.4%, *p* < 0.001).

**Conclusions:**

Compared to the general population, patients with SMI are at substantially increased risk of domestic and sexual violence, with a relative excess of family violence and adverse health impact following victimization. Psychiatric services, and public health and criminal justice policies, need to address domestic and sexual violence in this at-risk group.

## Introduction

Past research on violence and mental illness has focused on violence perpetrated by patients with psychotic disorders (Choe *et al.* 2008; Maniglio, 2009). The perception that people with severe mental illness (SMI) are dangerous is one of the key drivers of stigma against this group (Link *et al.* 1999). However, there is increasing evidence that violence against SMI patients is an important, under-researched public health problem (Choe *et al.* 2008).

Patients with SMI experience high rates of domestic and sexual violence, but the prevalence and health burden of these experiences compared with non-psychiatric controls is unknown (Hughes *et al.* 2012; Trevillion *et al.* 2012; Oram *et al.* 2013). In a recent systematic review of 42 studies, the median prevalence of adulthood domestic violence among female psychiatric patients was 30%, but no studies included control populations and there was little evidence on male victims, emotional abuse, and violence perpetrated by family members (other than partners) (Oram *et al.* 2013).

In the general population, domestic and sexual violence are a public health priority due to their significant morbidity and mortality; including injuries, chronic physical illness, poor sexual health, adverse perinatal outcomes, substance misuse, mental illness and suicidal behaviour (Ellsberg *et al.* 2008; Devries *et al.* 2013; WHO, 2013*a*). There is some evidence that the health burden is even greater among those with pre-existing disability (Khalifeh *et al.* 2013), but the health burden among people with SMI is unknown. Interventions are primarily based on evidence obtained from general population and primary-care samples (Feder *et al.* 2013; Taft *et al.* 2013), but findings may not generalize to psychiatric populations, where the nature and/or impact of violence may differ.

In order to address these key evidence gaps on the epidemiology of domestic and sexual violence against patients with SMI, we conducted a study which directly compared these outcomes among SMI patients and the general population. We tested the following hypotheses: (*a*) men and women with SMI would have increased odds of being victims of lifetime and past-year domestic and sexual violence compared to those without SMI, (*b*) family (non-partner) violence would comprise a greater proportion of domestic violence among victims with SMI than general population victims, (*c*) violence would lead to greater adverse health effects and less disclosure among victims with SMI than general population victims.

## Method

### Design

We recruited patients with SMI under the care of community mental health services using simple random sampling, and interviewed them using a modified version of the Crime Survey for England and Wales (CSEW) questionnaire (which includes an optional self-completion module on being a victim of domestic or sexual violence). We compared findings from our patient sample with findings from participants in the contemporaneous Office for National Statistics (ONS) cross-sectional crime survey (CSEW).

### Setting and participants

The patient sample was recruited from 19 community mental health teams (CMHTs) in two National Health Service (NHS) mental health organizations (Camden and Islington NHS Foundation Trust and South London and Maudsley NHS Foundation Trust). These Trusts serve a population of 1.5 million people living in a large diverse catchment area which includes pockets of deprivation and more affluent neighbourhoods. CMHTs serve people who require secondary mental healthcare, i.e. who have SMI (mainly affective and non-affective psychosis, but also severe non-psychotic mental disorders). Those requiring on-going care are assigned a named key-worker, who plans and coordinates their care. We used central IT registers to identify all patients with a named key-worker in the included teams, and drew a random sample from which we recruited our participants (for the period September 2011–March 2013). Inclusion criteria for patients were: (*a*) age 18–59 years, (*b*) under the care of CMHTs in one of six London boroughs for ⩾1 year, (*c*) living in the community (i.e. not in long-stay rehabilitation wards). Exclusion criteria were poor English-language proficiency and lack of capacity to consent. In this study, we included participants who completed the domestic/sexual violence module.

The comparison group was drawn from participants in the 2011–2012 ONS crime survey (CSEW). The CSEW recruited a nationally representative sample of people living in private residential households. One adult per household was recruited (drawn at random from the household's adult residents). For this study, the inclusion criteria for the comparison sample were: (*a*) aged 18–59 years, (*b*) completed the domestic/sexual violence module. We conducted an additional sensitivity analysis, where the comparison group was restricted to the subgroup of CSEW participants who fulfilled the above two criteria and lived in London.

After complete description of the study to potential participants, written informed consent was obtained.

### Interview procedures

The ONS national crime survey was conducted by lay interviewers in participants’ homes (TNS-BMRB, 2012). It comprised: (*a*) a computer-assisted face-to-face interview with all participants, which measured socio-demographics and experiences of past-year crime and (*b*) an opt-in computer-assisted self-completion questionnaire, which focused on the more sensitive topics of domestic and sexual violence. For the latter, participants were given a laptop, asked to enter the answers themselves, and assured that their responses would remain hidden from the interviewer. In the national crime survey, the self-completion module is typically completed by 70% of eligible respondents (TNS-BMRB, 2012).

The patient survey was conducted by one of six interviewers (three psychologists, one psychiatrist and two research assistants). One interviewer from each site attended ONS CSEW interviewer training and trained the others, in order to keep interview procedures as similar to the ONS survey as possible. Patients completed the modules pertaining to socio-demographics, crime victimization, domestic and sexual violence, safety perceptions, experiences with the criminal justice system and alcohol/drug use. As with the ONS survey, all patients were interviewed using a computer-assisted face-to-face interview, and were then invited to participate in the self-completion module. As with the CSEW, the opt-in module was computer-assisted, with the patients being given a laptop and asked to enter the answers themselves in private. For the minority of patients who did not want to do this, they were offered the option of either completing a paper-based questionnaire in private, or of having the questions read out to them by the interviewer. Where a paper-based version was used at the time of the interview, the interviewers entered the responses electronically shortly after the interview. All interviews were held in a quiet confidential location, in either a clinical setting or in the participant's home (depending on participant choice).

### Measures

The primary exposure was SMI, namely chronic mental illness requiring on-going care from secondary mental health services. In the study setting, the majority of such patients have affective or non-affective psychosis.

The main outcomes were: (1) being a victim of any domestic violence since the age of 16 and in the past year, (2) being a victim of any sexual violence since the age of 16 and in the past year. These outcomes were subdivided according to (*a*) the nature of violence, (*b*) the perpetrator, as detailed in Table 1. Sexual violence perpetrated by partners or family members was included in the definitions of both domestic violence and sexual violence, in accordance with international definitions (WHO, 2013*b*).

**Table 1.** Definition of outcomes

***Domestic violence***: Emotional, physical or sexual abuse (as defined below) perpetrated by *partner* (boyfriend or girlfriend; husband, wife or civil partner) or *family* member other than partner (parents, children, siblings or any other relatives).
***Emotional abuse***: perpetrator did any of the following: (*a*) Prevented them from having fair share of money. (*b*) Stopped them from seeing friends or relatives. (*c*) Repeatedly belittled them so they felt worthless. (*d*) Threatened to hurt them or someone close to them. (*e*) Threatened them with a weapon or threatened to kill them.
***Physical violence***: perpetrator did any of the following: (*a*) Pushed them, held them down or slapped them. (*b*) Kicked, bit or hit them, or threw something at them. (*c*) Choked or tried to strangle them. (*d*) Used some other kind of force against them.
***Sexual violence***: perpetrator did any of the following in a way that caused fear, alarm or distress: (*a*) Indecently exposed themselves to them. (*b*) Touched them sexually when they did not want it (e.g. groping, touching of breasts or bottom, unwanted kissing). (*c*) Forced them to have sexual intercourse, or to take part in some other sexual act, when they made it clear that they did not agree or when they were not capable of consent (*serious sexual assault*). We divided sexual violence by perpetrator into *sexual domestic violence* (perpetrated by partner or family members) and *sexual non-domestic violence* (perpetrated by strangers or acquaintances). The control study sample was randomly divided into two groups with slightly different questions on the perpetrator of sexual violence- such that it was possible to estimate domestic sexual violence in the whole study sample, and non-domestic sexual violence in only half the sample. We were able to estimate these subtypes for the entire patient sample.
***Adverse impact of serious sexual assaults (SSA)***: SSA led to one or more of the following: (*a*) Physical injuries/illness: minor bruising or black eye, scratches, severe bruising or bleeding from cuts, internal injuries or broken bones/teeth, other physical injuries, contracting a disease, becoming pregnant. (*b*) Psychological/social problems: mental or emotional problems, such as difficulty sleeping/nightmares; depression; low self-esteem; stopped trusting people/difficulty in other relationships; stopped going out. (*c*) Suicide attempt.

The following additional outcomes were only asked about among victims of serious sexual assault (SSA) (i.e. rape or attempted rape) since the age of 16: (1) impact, measured by asking victims if they had experienced any of the following as a result of SSA: (1a) physical illness or injury, (1b) psychosocial problems or (1c) suicide attempts (see Table 1 for details); (2) reporting of SSA (to professionals or informal social networks).

Potential socio-demographic confounders, which were identified *a priori* from the literature, were: age, sex, ethnicity, educational attainment, employment, lone adult in household, child(ren) in household, housing tenure and small area multiple deprivation index (Walby & Allen, 2004; Abramsky *et al.* 2011;ONS, 2013). We explored the potential mediating effect of substance misuse (measured as frequency of drunkenness in the past year, and any past illicit drug use).

Clinical diagnosis was defined as the primary ICD-10 diagnosis recorded in the electronic clinical records.

### Statistical analysis

All analyses were performed using Stata v. 12 (StataCorp., USA). Since we wished to examine outcomes in both men and women, all analyses were stratified by gender. We estimated odds ratios (ORs) for domestic and sexual violence among patients with SMI compared with general population controls using multivariate logistic regression. We entered covariates in three sequential blocks (model 1: age only; model 2: add other socio-demographics; model 3: add substance misuse) to explore to what extent these domains accounted for any excess violence risk. We interpreted the latter with caution, since adjusting for potential mediators may bias the main-effect estimates (Robins & Greenland, 1992; Hernandez Diaz *et al.* 2006).

We compared health impact and disclosure of SSA among victims with and without SMI using *χ*^2^ tests.

Past literature had suggested that the gender gap seen in the general population (with excess risk for women for domestic and sexual violence) was attenuated among people with SMI (Khalifeh & Dean, 2010). To explore this, we estimated the crude and adjusted ORs for domestic and sexual violence in women compared to men among patients with SMI and then among general population controls.

We conducted a sensitivity analysis, comparing adulthood and past-year domestic and sexual violence against the patient sample compared to a subgroup of CSEW participants who lived in London.

## Results

Note that all reported ‘adjusted odds ratios’ below refer to ORs adjusted for socio-demographics (model 2 in Tables 3 and 4). We comment separately on ORs additionally adjusted for substance misuse (model 3 in Tables 3 and 4).

### Sample flow and characteristics

We recruited 361/697 eligible patients (52% response rate). Of the 345 participants aged 18–59 years, 303 (88%) took part in the self-completion module on domestic/sexual violence; non-respondents did not differ from respondents in terms of age, sex or educational attainment (data not shown). In total, 46 031 people participated in the 2011/12 ONS CSEW survey (72% response rate). Of the 28 324 participants aged 18–59 years, 22 606 (80%) took part in the self-completion module on domestic/sexual violence; non-respondents were more likely to be older and unemployed.

Table 2 shows sample characteristics. Patients with SMI had greater levels of social deprivation than the comparison group. Sixty percent (*n* = 181) had a diagnosis of schizophrenia and 53% (*n* = 162) had a history of involuntary admission to hospital.

**Table 2.** Sample characteristics

Socio-demographics	Patients (total *N* = 303) *n* (%)	Controls (total *N* = 22 606) *n* (%)	*p* value for patients *v.* controls
Age, years, mean (s.d.)	40.8 (0.58)	39.4 (11.3)	0.04
Sex			<0.001
Male	170 (56.1)	10318 (45.6)	
Female	133 (43.9)	12288 (54.4)	
Ethnicity			<0.001
White	124 (40.9)	20499 (90.7)	
Black/Black British	72 (23.8)	1504 (6.7)	
Asian/Chinese/Other	106 (35.0)	592 (2.6)	
Marital status		<0.001	
Single	224 (73.9)	9029 (39.9)	
Married	22 (7.3)	10098 (44.7)	
Divorced/widowed	52 (17.2)	3474 (15.4)	
Never had partner	29 (9.6)	303 (1.3)	<0.001
Living alone	208 (68.6)	5947 (26.3)	<0.001
Children in household	36 (11.9)	9238 (40.9)	<0.001
Employment status		<0.001	
Employed	32 (10.6)	17909 (79.2)	
Student/economically inactive	29 (9.6)	2589 (11.5)	
Long-term sick/unemployed	242 (79.9)	2085 (9.2)	
Tenancy			<0.001
Owner	18 (5.9)	13933 (61.6)	
Rents private flat	88 (29.0)	5453 (24.1)	
Rents council flat (state-funded)	196 (64.7)	3179 (14.1)	
Drunk ⩾1 once/month	49 (16.2)	2275 (10.1)	0.05
Illicit drug use past year	102 (33.7)	1684 (7.4)	<0.001
Clinical characteristics			
Diagnosis			
Schizophrenia and related disorders	181 (59.7)		
Bipolar affective disorder	35 (11.6)		
Recurrent depressive disorder	30 (9.9)		
Personality disorder	23 (7.6)		
Other	34 (11.2)		
History of involuntary hospital admission	162 (53.5)		
Number of hospital admissions, mean (s.d.)	3 (3.5)		
Illness duration, mean (s.d.)	13 (8.9)		

Values given are *n* (%) unless stated otherwise.

### Domestic violence: prevalence and relative odds (Table 3 and Fig. 1)

Comparing SMI patients with controls, adulthood domestic violence was reported by 69% *v.* 33% of women [OR adjusted for socio-demographics (aOR) 3.9, 95% confidence interval (CI) 2.6–5.8] and 49% *v.* 17% of men (aOR 3.5, 95% CI 2.5–5.1), respectively.

**Fig. 1.**
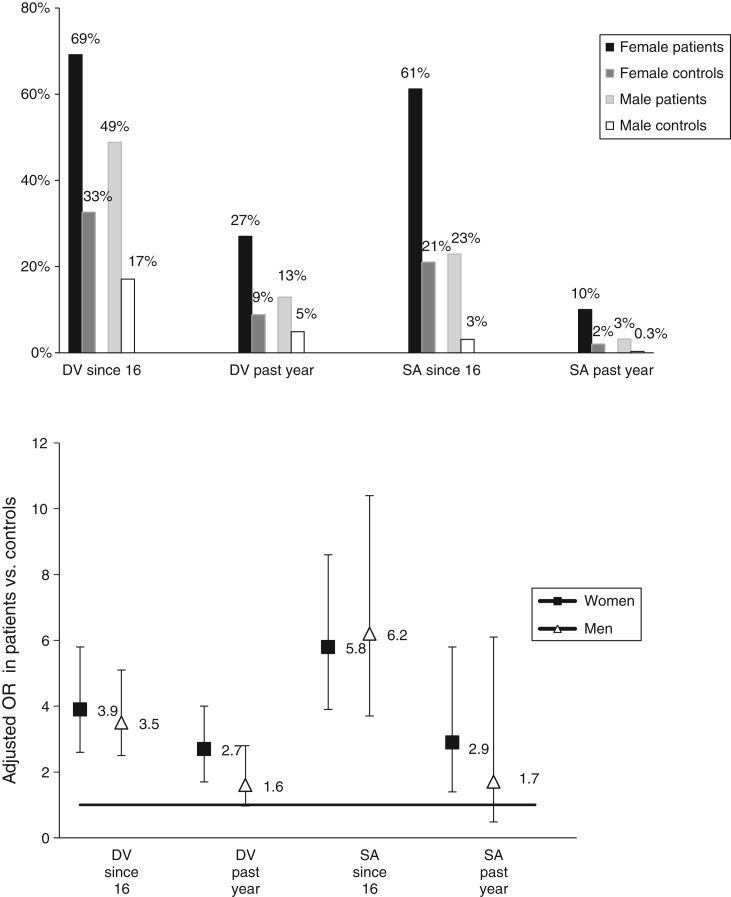
Prevalence and adjusted odds for domestic violence (DV) and sexual assault (SA) victimization.

**Table 3.** Prevalence and odds of adulthood and past-year domestic violence (DV) among patients and controls, by gender

	Prevalence				Relative odds								
	Patients		Controls		Model 1^b^			Model 2^c^			Model 3^d^		
	Total *N*^a^	*n* victims (%)	Total *N*^a^	*n* victims (%)	OR	95% CI	*p*	OR	95% CI	*p*	OR	95% CI	*p*
Women													
Any DV since age 16	133	92 (69.2)	12288	4007 (32.6)	4.6	3.2–6.7	<0.001	3.9	2.6–5.8	<0.001	3.4	2.2–5.3	<0.001
Emotional	133	84 (63.2)	12288	3293 (26.8)	4.6	3.3–6.6	<0.001	3.9	2.7–5.8	<0.001	3.5	2.3–5.4	<0.001
Physical	133	79 (59.4)	12288	2841 (23.1)	4.8	3.4–6.8	<0.001	4.1	2.8–6	<0.001	3.2	2.1–4.9	<0.001
Sexual	133	37 (27.8)	12288	887 (7.2)	4.9	3.3–7.3	<0.001	3.3	2.1–5.3	<0.001	2.7	1.6–4.4	<0.001
Partner	124	77 (62.1)	12164	3613 (29.7)	2.8	2.1–3.7	<0.001	3.2	2.1–4.7	<0.001	2.6	1.7–4.0	<0.001
Family	133	56 (42.1)	12288	1269 (10.3)	6.4	4.5–9.1	<0.001	3.4	2.3–5.1	<0.001	3.2	2.1–4.8	<0.001
Any DV in past year	133	36 (27.1)	12288	1085 (8.8)	3.8	2.6–5.5	<0.001	2.7	1.7–4.0	<0.001	2.4	1.5–3.9	<0.001
Partner	124	21 (16.9)	12164	890 (7.3)	2.6	1.6–4.2	<0.001	1.8	1.1–3.1	<0.01	1.7	0.95–2.9	0.08
Family	133	21 (15.8)	12288	336 (2.7)	7.1	4.4–11.6	<0.001	3.4	1.9–6	<0.001	3.1	1.7–5.9	<0.001
Men													
Any DV since age 16	170	83 (48.8)	10318	1763 (17.1)	4.5	3.3–6.1	<0.001	3.5	2.5–5.1	<0.001	3.3	2.3–4.9	<0.001
Emotional	170	73 (42.9)	10318	1295 (12.6)	5.1	3.7–7.0	<0.001	3.2	2.2–4.6	<0.001	3	2.1–4.5	<0.001
Physical	170	54 (31.8)	10318	1091 (10.6)	3.8	2.7–5.3	<0.001	3.5	2.4–5.3	<0.001	3.3	2.1–5	<0.001
Sexual^e^	170	7 (4.1)	10318	61 (0.6)	–	–	–	–	–	–	–	–	−
Partner	149	57 (38.3)	10138	1426 (14.1)	3.7	2.6–5.1	<0.001	2.8	1.9–4.2	<0.001	2.7	1.8–4.1	<0.001
Family	170	54 (31.8)	10318	726 (7)	6.5	4.6–9.0	<0.001	3.6	2.4–5.4	<0.001	3.3	2.2–5.1	<0.001
Any DV in past year	170	22 (12.9)	10318	507 (4.9)	2.9	1.8–4.6	<0.001	1.6	0.97–2.8	0.07	1.4	0.83–2.5	0.19
Partner	149	14 (9.4)	10138	390 (3.8)	2.6	1.5–4.5	<0.01	1.5	0.82–2.9	0.18	1.3	0.65–2.5	0.48
Family	170	11 (6.5)	10318	175 (1.7)	4.2	2.2–7.9	<0.001	1.5	0.71–3	0.29	1.3	0.6–2.8	0.50

OR, Odds ratio; CI, confidence interval.

a Total *N* for partner violence excluded participants who had never had a partner.

b Model 1: Adjusted for age.

c Model 2: Adjusted for age, ethnicity, marital status, living alone, having children, employment, housing tenure, area deprivation.

d Model 3: Adjusted for factors in model 2, and additionally frequency of drunkenness in past year and any past-year illicit drug use.

e Absolute numbers in patients were too low to allow for stable estimates.

The relative adjusted odds for each of the different forms of lifetime DV (emotional/physical/sexual; and partner/family) were elevated around 3- to 4-fold among both men and women with SMI at the 5% significance level. Absolute number for sexual domestic violence in men was too low for stable estimates. Past-year domestic violence was reported by 27% *v.* 9% of women (aOR 2.7, CI 1.7–4.0) and 13% *v.* 5% of men (aOR 1.6, 95% CI 1.0–2.8) with and without SMI, respectively, with elevated odds for both partner and family violence among women with SMI (further detailed analyses are reported in Table 3).

Among victims of domestic violence, a greater proportion of victims with SMI than control victims experienced family violence (61% *v.* 32% among women and 65% *v.* 41% among men; *p* < 0.001) (online Supplementary Table S1).

### Sexual assaults: prevalence and relative odds (Table 4 and Fig. 1)

Comparing SMI patients with controls, adulthood sexual assaults were reported by 61% *v*. 21% of women (aOR 5.8, 95% CI 3.9–8.6) and 23% *v.* 3% of men (aOR 6.2, 95% CI 3.7–10.4), respectively. Adulthood serious SSA were reported by 40% *v.* 7% of women (aOR 6.2, 95% CI 4.1–9.6) and 12% *v.* 0.5% of men (aOR 7.8, 95% CI 3.6–16.9), respectively. Past-year sexual assaults were reported by 10% *v.* 2% of women (aOR 2.9, 95% CI 1.4–5.8). Absolute numbers among men were too low to allow for stable estimates.

**Table 4.** Prevalence and odds of adulthood and past-year sexual assaults (SA) among patients and controls, by gender

	Prevalence				Relative odds								
	Patients		Controls		Model 1^b^			Model 2^c^			Model 3^d^		
	Total *N*	*N* victims (%)	Total *N*^a^	*N* victims (%)	OR	95% CI	*p*	OR	95% CI	*p*	OR	95% CI	*p*
Women													
Any SA since age 16	129	79 (61.2)	12289	2587 (21.1)	5.9	4.1–8.5	<0.001	5.8	3.9–8.6	<0.001	4.4	2.9–6.8	<0.001
Indecent exposure	129	45 (34.9)	12289	1316 (10.7)	5.2	3.6–7.6	<0.001	5.5	3.5–8.4	<0.001	4.2	2.6–6.7	<0.001
Unwanted sexual touching	129	56 (43.4)	12289	1567 (12.8)	5.6	4.1–8.6	<0.001	4.9	3.3–7.4	<0.001	3.6	2.3–5.6	<0.001
Serious sexual assaults	129	52 (40.3)	12289	871 (7.1)	9.3	6.4–13.3	<0.001	6.2	4.1–9.6	<0.001	4.8	3.0–7.7	<0.001
Domestic SA	129	37 (28.7)	6117	396 (6.5)	5.8	3.9–8.6	<0.001	4.6	2.8–7.7	<0.001	3.7	2.1–6.5	<0.001
Non-domestic SA	129	61 (47.3)	6117	907 (14.8)	5.2	3.6–7.4	<0.001	6.6	4.3–10.2	<0.001	5.2	3.2–8.3	<0.001
Any SA in past year	129	13 (10.1)	12288	245 (2.0)	6	3.3–10.9	<0.001	2.9	1.4–5.8	<0.01	2.1	0.98–4.7	0.05
Men													
Any SA since age 16	157	36 (22.9)	10317	321 (3.1)	9.3	6.3–13.7	<0.001	6.2	3.7–10.4	<0.001	5.5	3.2–9.5	<0.001
Indecent exposure	157	12 (7.6)	10317	129 (1.3)	6.7	3.6–12.4	<0.001	4.8	2.1–10.7	<0.001	4.5	1.9–10.5	<0.001
Unwanted sexual touching	157	26 (16.6)	10317	193 (1.9)	10.9	6.9–17.0	<0.001	7.1	3.8–13.0	<0.001	6.2	3.2–11.8	<0.001
Serious sexual assaults	157	19 (12.1)	10317	56 (0.5)	24.4	14.1–42.4	<0.001	7.8	3.6–16.9	<0.001	6.3	2.8–14.2	<0.001
Domestic SA^e^	157	7 (4.5)	5195	18 (0.35)	–	–	–	–	–	–	–	–	–
Non-domestic SA	157	32 (20.4)	5195	107 (2.1)	12.6	8.1–19.4	<0.001	12.4	5.9–25.7	<0.001	10.9	5.0–23.7	<0.001
Any SA in past year^e^	157	5 (3.2)	10317	33 (0.32)	–	–	–	–	–	–	–	–	–

a Perpetrator of sexual assaults was asked about in all patient participants but only a random half of control participants; reflected in total *N* for domestic and non-domestic SA.

b Model 1: Adjusted for age.

c Model 2: Adjusted for age, ethnicity, marital status, living alone, having children, employment, housing tenure, area deprivation.

d Model 3: Adjusted for factors in model 2, and additionally frequency of drunkenness in past year and any past-year illicit drug use.

e Absolute numbers in patients were too low to allow for stable estimates.

The proportion of sexual assaults by perpetrator is shown in online Supplementary Table 1.

### The effect of adjusting for substance misuse (Tables 3 and 4)

The adjustment for substance misuse in addition to socio-demographics resulted in a reduction in the ORs by 4–22% for domestic violence and 6–26% for sexual assaults. ORs at the 5% significance level remained elevated for lifetime and past-year violence, apart from past-year domestic violence in men (aOR 1.4, CI 0.82–2.5).

### SSA: impact and reporting (Table 5)

These outcomes were only estimated for female victims of SSA, as the absolute number of male victims was too low for stable estimates. Compared to female victims without SMI, victims with SMI were more likely to report adverse psychological/social effects (92% *v.* 64%, *p* < 0.001) and attempted suicide (53% *v.* 3%, *p* < 0.001) as a result of experiencing SSA, but equally likely to report physical illness or injury (49% *v.* 40%, *p* = 0.35) as a result of experiencing SSA. Women with SMI who were victims were more likely than control victims to disclose their experiences to health professionals (43% *v.* 15%, *p* < 0.001) and to the police (37% *v.* 16%, *p* < 0.001), but a similar proportion disclosed to informal networks in the two groups.

**Table 5.** Serious sexual assaults: frequency of adverse effects and disclosure among patient and control female victims

	Patients		Controls		*p* value for patients *v.* controls
	Total *N* victims	*N* victims reporting consequence/disclosure	Total *N* victims	*N* victims reporting consequence/disclosure	
Consequences of serious sexual assaults					
Any adverse impact	95.9	47 (95.9)	827	648 (78.4)	<0.01
Physical injuries/disease	49	24 (49)	827	334 (40.4)	0.35
Psychological/social	91.8	45 (91.8)	827	531 (64.2)	<0.001
Suicide attempt	53.1	26 (53.1)	827	28 (3.4)	<0.001
Disclosure of serious sexual assaults					
To anyone	75.5	37 (75.5)	827	481 (58.2)	0.02
Friends/relatives/neighbours	51	25 (51)	827	387 (46.8)	0.86
Health professional (e.g. doctor, nurse, mental health social worker, etc.)	42.9	21 (42.9)	827	127 (15.4)	<0.001
Police	36.7	18 (36.7)	827	129 (15.6)	<0.001
Other	22.4	11 (22.4)	827	164 (19.8)	0.76

### Gender and risk of domestic and sexual violence

Among both patients and controls, women had around 6- to 9-fold elevated odds of being victims of sexual violence, 2- to 3-fold elevated odds of partner violence, and 30–40% elevated odds of family violence (the latter did not meet statistical significance at 5% level among patients) (online Supplementary Table S2).

### Sensitivity analysis

The results of sensitivity analyses, which compared adulthood and past-year domestic and sexual violence in the patient sample with London-based controls, are shown in online Table S3. The adjusted ORs were 5–9 times higher among women with SMI, and 4–7 times higher among men with SMI (with wide CIs, but all exceeding 1 at the 95% significance level). Online Supplementary Fig. S1 summarizes the adjusted ORs from the analyses comparing the patient sample with both London-based controls and national-based controls. The point estimates for the adjusted ORs were higher in London-based comparisons, but the CIs were wide and overlapped with those from national-based comparisons.

## Discussion

This study compared the prevalence of domestic and sexual violence against patients with SMI under the on-going care of mental health services with a general population control group, and found a high prevalence and markedly excess odds of these experiences among patients with SMI. Among domestic violence victims, family violence was experienced by a greater proportion of SMI than control victims. Women with SMI were more likely to attempt suicide as a result of SSA than female victims without SMI, and more likely to disclose sexual violence to health professionals and the police.

The prevalence estimates for domestic and sexual violence among women with SMI are in line with previous studies (Goodman *et al.* 1997; Teplin *et al.* 2005; Hughes *et al.* 2012). To our knowledge, no past studies have compared domestic violence in psychiatric patients with a general population control sample (Oram *et al.* 2013). We found that people with diagnosed SMI in contact with psychiatric services had 2- to 4-fold elevated odds of all subtypes of domestic violence (emotional, physical and sexual) compared to the general population. These findings suggest that clinicians should routinely enquire not just about physical domestic violence, but also emotional and sexual abuse – especially given the increasing evidence that emotional abuse may have a greater health impact than physical violence (Yoshihama *et al.* 2009; Jewkes, 2010). The relationship between experiencing violence and SMI is likely to be bi-directional (Danielson *et al.* 1998; Chen *et al.* 2010; Jonas *et al.* 2014), but we report increased risk of recent violence occurring after illness onset. In this study, substance misuse appeared to account for a proportion of the excess violence risk, and may be a suitable target for intervention, although the direction of causality is unclear, since being a victim can lead to increased substance misuse as a coping mechanism (Coker *et al.* 2002).

We found that family violence comprised a greater proportion of overall domestic and sexual violence experiences among victims with SMI than general population victims (Krug, 2002). People with SMI are known to have elevated risks of childhood maltreatment, and abuse by family members, including parents, may extend into adulthood (Varese *et al.* 2012). Most domestic violence prevention policies among working-age adults have focused on partner violence, but our findings suggest that interventions among patients with SMI also need to target family violence.

We detected a 6- to 8-fold elevation in the odds of sexual assault among both men and women with SMI. This is lower than the 17-fold risk reported in a recent US study (Teplin *et al.* 2005), but we adjusted for a broader range of confounders, and included estimates for lifetime rather than just past-year sexual assaults (where prevalence is low and estimates are imprecise). Half of the women with SMI who experienced SSA reported attempting suicide as a result of these experiences. In patients with SMI, suicide attempts may be seen as a direct result of acute psychotic relapse (Fialko *et al.* 2006), with under-detection of trauma and related post-traumatic stress disorder as a trigger for suicidal behaviour.

The finding of substantially elevated risk of domestic and sexual violence victimization among patients with SMI mirrors the findings of a high prevalence of all types of victimization, including violent crime by strangers or acquaintances (Bengtsson-Tops & Ehliasson, 2012; Katsikidou *et al.* 2013), as well as non-violent crime such as thefts, burglaries and criminal damage (Teplin *et al.* 2005). Future research should explore shared and unique risk factors for these victimization experiences, in order to guide effective interventions. Patients with an abuse history may benefit from trauma-focused psychological therapy (Warshaw *et al.* 2013; WHO, 2013*b*). These interventions have an evidence base in non-psychiatric populations, mainly in antenatal or accident and emergency settings, but their effectiveness for patients with SMI has not been fully explored (Mueser *et al.* 2008).

Among victims of sexual assault, a higher proportion of SMI than control victims reported their experiences to the police, but there is evidence that they are often disbelieved and discriminated against within the criminal justice system (Hester, 2013; Pettit *et al.* 2013). Only 43% of patients had disclosed their experiences to a healthcare professional, despite the fact that this patient population had received intensive support from psychiatric services for at least a year in order to be included in the study. Health professionals often fail to detect trauma histories in patients with SMI, or where they do detect it, they often fail to address it in patients’ management plans, (Howard *et al.* 2010; Nyame *et al.* 2013). This may lead to treatment resistance for the primary mental disorder (Mueser *et al.* 2002). There is therefore a need for interventions that improve detection of violence by healthcare professionals, and the provision of subsequent support. There is evidence from a pilot study that a complex intervention which includes reciprocal training of mental health and domestic violence sector professionals, and a care pathway with integrated advocacy services, can improve detection and outcomes of domestic violence among psychiatric patients (Trevillion *et al.* 2014). Our findings suggest the need to include screening and support for sexual assaults in such interventions. Effective interventions would require joint working with voluntary sector organization and the criminal justice system (Krug, 2002; WHO, 2013*b*).

Strengths of this study include: the large randomly selected sample; reliable, validated measures of violence experiences; hypothesis-based analyses and careful adjustment for confounders. We adjusted for a broader range of confounders than most previous related studies (Hughes *et al.* 2012; Oram *et al.* 2013), including adjustment for demographics and individual/area deprivation. We also explored potential mediation by substance misuse. One limitation is the lack of data on violence perpetration among controls, so we could not adjust for the potential mediating effect of this factor.

Potential limitations include the cross-sectional nature of the study, which precludes firm conclusions about direction of causality. All patients had been under the care of mental health services for more than 1 year, so by definition past-year violence would have occurred after the onset of SMI (notwithstanding measurement error). Nonetheless the causal direction remains uncertain, since patients with SMI may have had historical victimization experiences, which may put them at risk of recent violence.

The response rate was somewhat low at 52%, but we researched a sensitive topic in a hard-to-reach population. Although domestic and sexual violence are sensitive topics for any group, they may be even more sensitive and complex for patients in secondary mental healthcare to discuss. This is because this particular group suffers from stigma related to violence risk (Link *et al.* 1999), and may worry about additional consequences of disclosure such as involuntary hospital admission (Pettit *et al.* 2013). We used a rigorous random sampling procedure rather than a convenience sample (unlike many previous related studies) (Hughes *et al.* 2012; Oram *et al.* 2013), and non-responders had the same demographic profile (in terms of age and sex) as participants. We did not have additional details on the characteristics of non-responders, so it is difficult to comment on the likely magnitude and direction of non-response bias.

It is worth noting that this study relates to patients with SMI in contact with secondary mental health services, so the findings may not generalize to those with similar mental disorders who do not require on-going psychiatric care. In national UK surveys, two thirds of patients with a diagnosis of a psychotic disorder were found to be in contact with mental health services (McManus *et al.* 2010). Those in contact with services may be at increased risk of victimization, due to a potential excess of risk factors such as social isolation, substance misuse or violence perpetration.

The crime survey definition of domestic violence does not have sufficient detail on context, severity and frequency to allow a distinction between recurrent, controlling severe abuse and incidents of violence reflecting relationship couple tension (Johnson, 2006). Reporting bias is possible, since patients and controls may have different thresholds for disclosing violence, although there is no evidence to suggest that people with SMI over-report these experiences (Goodman *et al.* 1999). Residual confounding is possible. This general population control sample may have included a small proportion of people with SMI (<3%) (Health and Social Care Information Centre, 2013) although the effect of this would have been to have biased the ORs closer to the null. We compared a London-based patient sample with a national control sample (to ensure adequate power), but violence prevalence did not differ by region of residence in the control group (ONS, 2013). The findings from the sensitivity analysis, which compared patients to London-based controls, were consistent with those comparing patients to national-based controls.

## Conclusion

Men and women with SMI who are under the on-going care of psychiatric services are 2–8 times more likely to experience sexual and domestic violence than the general population, with a high relative burden of family violence. Women with SMI are more likely than women in the general population to suffer psychological ill health and attempt suicide following sexual assaults, but most do not disclose violence to healthcare professionals. Healthcare professionals need to work closely with the voluntary sector and criminal justice system in order to effectively address the high burden of violence in this population. Potentially effective support includes advocacy and trauma-focused psychological interventions (Mueser *et al.* 2008; Trevillion *et al.* 2014). Healthcare professionals need to consider victimization as a potential trigger for suicide attempts among patients. Future research should explore reasons for non-disclosure to healthcare professionals, and test the effectiveness of interventions to improve the detection of victimization and support offered by mental healthcare professionals.

## Supplementary material

For supplementary material accompanying this paper visit http://dx.doi.org/10.1017/S0033291714001962. click here to view supplementary material
