# Supplementary material for: Domestic and sexual violence against patients with severe mental illness
Source: Psychol Med. 2014 Sep 4;45(4):875–86. doi: 10.1017/S0033291714001962 (PMC4413870; doi:10.1017/S0033291714001962)
Supplement: Supplementary file 1 [file S0033291714001962sup001.docx]

**SUPPLEMENTARY MATERIAL**

Supplementary Table 1 Perpetrators of adulthood domestic and sexual violence among patient and control victims

|  | Patients | | | | | | Controls | | | P for difference between patients and controls |
| --- | --- | --- | --- | --- | --- | --- | --- | --- | --- | --- |
|  | Total N who reported any DV | | | | Total N who reported DV by this perpetrator | % | Total N who reported any DV | Total N who reported DV by this perpetrator | % |  |
| **DOMESTIC VIOLENCE** | | | | |  |  |  |  |  |  |
| **Perpetrator of DV against women** | | | |  |  |  |  |  |  |  |
| Partner | | 92 | | | 77 | 83.7 | 4007 | 3613 | 90.2 | 0.04 |
| Family member | | 92 | | | 56 | 60.9 | 4007 | 1269 | 31.7 | <0.001 |
| Partner & family member | | 92 | | | 41 | 44.6 | 4007 | 875 | 21.8 | <0.001 |
| **Perpetrator of DV against men** | |  | | |  |  |  |  |  |  |
| Partner | | 83 | | | 57 | 68.7 | 1763 | 1426 | 80.9 | <0.01 |
| Family member | | 83 | | | 54 | 65.1 | 1763 | 726 | 41.2 | <0.001 |
| Partner & family member | | 83 | | | 28 | 33.7 | 1763 | 389 | 22.1 | 0.01 |
| **SEXUAL ASSAULTS** | | | | |  |  |  |  |  |  |
| **Perpetrator of sexual assault against women** | |  | | |  |  |  |  |  |  |
| Partner | | 79 | | | 32 | 40.5 | 1173 | 348 | 29.7 | 0.04 |
| Family member | | 79 | | | 12 | 15.2 | 1173 | 73 | 6.2 | <0.01 |
| Acquaintance | | 79 | | | 34 | 43.0 | 1173 | 339 | 28.9 | <0.01 |
| Stranger | | 79 | | | 48 | 60.8 | 1173 | 656 | 55.9 | 0.4 |
| **Perpetrator of sexual assault against men** | | |  | |  |  |  |  |  |  |
| Partner or family member^2^ | | 36 | | | 7 | 19.4 | 123 | 18 | 14.6 | 0.49 |
| Acquaintance | | 36 | | | 20 | 55.6 | 123 | 54 | 43.9 | 0.22 |
| Stranger | | 36 | | | 16 | 44.4 | 123 | 57 | 46.3 | 0.84 |

1. Perpetrator of sexual assaults was asked about in all patient participants but only a random half of control participants
2. Absolute numbers of partner and family member perpetrators were too low to report separately

Supplementary Table 2 Relative odds for being the victim of partner, family and sexual violence since age 16 in women compared with men (assessed separately in patients and controls)

|  | **Prevalence** | | | | **Odds ratios in women vs. men** | | | | | |
| --- | --- | --- | --- | --- | --- | --- | --- | --- | --- | --- |
|  | Women | | Men | | Odds ratio  (model 1)^1^ | 95% CI | p | Odds ratio  (model 2)^2^ | 95% CI | p |
|  | N | n | N | n |  |  |  |  |  |  |
| **Patients** |  |  |  |  |  |  |  |  |  |  |
| Partner violence | 133 | 77 | 169 | 57 | 2.7 | 1.7-4.4 | <0.001 | 3.0 | 1.7-5.4 | <0.001 |
| Family violence | 133 | 56 | 169 | 54 | 1.5 | 0.93-2.4 | 0.09 | 1.3 | 0.77-2.3 | 0.09 |
| Sexual violence | 133 | 79 | 169 | 36 | 5.6 | 3.3-9.5 | <0.001 | 6.7 | 3.6-12.5 | <0.001 |
| **Controls** |  |  |  |  |  |  |  |  |  |  |
| Partner violence | 12,288 | 3611 | 10,318 | 1426 | 2.6 | 2.4-2.8 | <0.001 | 2.4 | 2.2-2.6 | <0.001 |
| Family violence | 12,288 | 1268 | 10,318 | 726 | 1.5 | 1.4-1.7 | <0.001 | 1.5 | 1.3-1.7 | <0.001 |
| Sexual violence | 12,288 | 2587 | 10,318 | 321 | 8.4 | 7.4-9.4 | <0.001 | 8.6 | 7.6-9.8 | <0.001 |

1. Model 1: Adjusted for age
2. Model 2: Adjusted for age, ethnicity, marital status, living alone, having children, employment, housing tenure, area deprivation

Supplementary Table 3 Prevalence and odds of adulthood and past-year domestic violence (DV) and sexual assaults (SA) among patients and London-based controls, by gender

|  | **Prevalence** | | | | | **Relative odds** | | | | | |
| --- | --- | --- | --- | --- | --- | --- | --- | --- | --- | --- | --- |
|  | Patients | | | Controls | | Model 1^a^ | | | Model 2^b^ | | |
|  | Total N | N victims (%) | | Total N | N victims (%) | OR | 95% CI | p | OR | 95% CI | p |
| WOMEN |  |  | |  |  |  |  |  |  |  |  |
| Any DV since 16 | 133 | 92 (69.2) | | 1142 | 292 (25.6) | 6.6 | 4.4-9.8 | <0.001 | 6.4 | 3.8-10.7 | <0.001 |
| Any DV in past year | 133 | 36 (27.1) | | 1142 | 57 (5.0) | 7.8 | 4.9-12.7 | <0.001 | 7.2 | 3.6-14.5 | <0.001 |
| Any SA since 16 | 129 | 79 (61.2) | | 1142 | 264 (23.1) | 5.1 | 3.5-7.5 | <0.001 | 8.7 | 5.1-14.8 | <0.001 |
| Any SA in past year | 129 | 13 (10.1) | | 1142 | 28 (2.5) | 5.1 | 2.5-10.4 | <0.001 | 5.1 | 1.8-14.6 | <0.001 |
| MEN |  |  | |  |  |  |  |  |  |  |  |
| Any DV since 16 | 170 | 83 (48.8) | | 1006 | 133 (13.2) | 6.2 | 4.3-8.8 | <0.001 | 5.4 | 3.1-9.6 | <0.001 |
| Any DV in past year | 170 | 22 (12.9) | | 1006 | 43 (4.3) | 3.4 | 1.9-5.9 | <0.001 | 4.2 | 1.7-10.2 | <0.001 |
| Any SA since 16 | 157 | | 36 (22.9) | 1006 | 35 (3.5) | 8 | 4.8-13.3 | <0.001 | 6.7 | 2.8-16 | <0.001 |
| Any SA in past year^d^ | - | - | | - | - | - | - | - | - | - | - |

1. Model 1: Adjusted for age
2. Model 2: Adjusted for age, ethnicity, marital status, living alone, having children, employment, housing tenure, area deprivation
3. Absolute numbers in patients and controls were too low to allow for stable estimates

Supplementary Figure 1
